# Effect of atrial septal dilation tumor and patent foramen rotundus on cryptogenic ischemic stroke in young and middle-aged patients

**DOI:** 10.4314/ahs.v22i3.35

**Published:** 2022-09

**Authors:** Tengtao Ling, Dongxing Xie

**Affiliations:** Department of Echocardiograhy, Tangshan Worker Hospital, Tangshan 063000, China

**Keywords:** Atrial septal aneurysm, Cryptogenic ischemic stroke, Influencing factors, Patent foramen ovale, Young and middle-aged

## Abstract

**Objective:**

To investigate the effect of atrial septal aneurysm (ASA) and patent foramen ovale (PFO) on cryptogenic ischemic stroke in young and middle-aged patients.

**Methods:**

A case-control study was conducted to select 96 young and middle-aged patients with cryptogenic ischemic stroke in the Department of Neurology of our hospital from January 2015 to January 2020 (observation group). A total of 192 patients with non-ischemic stroke were selected as the control group. The clinicadata and transthoracic echocardiography (cTTE) results were compared between the two groups.

**Results:**

The ASA depth and tumor wall thickness in the observation group were 15.50 (12.40, 19.80) mm and 3.90 (2.80,4.50) mm, respectively, which were significantly higher than those in the control group (P < 0.05). Logistic regression analysis showed that PFO, and PFO combined with ASA were the influencing factors of cryptogenic ischemic stroke in middle-aged and young people OR = 1.923 and 1.384, P < 0.05).

**Conclusion:**

Atrial septal aneurysm combined with PFO and PFO are independent influencing factors factor for the occurrence of cryptogenic ischemic stroke in young and middle-aged people, which is worthy of further study.

## Introduction

Some local ischemic strokes that cannot find a clear cause in clinic are called cryptogenic ischemic strokes, which occur frequently in young and middle-aged people. The etiology is not clear, and there are certain limitations in the treatment of cryptogenic ischemic stroke^1^. Studies have shown that patent foramen ovale (PFO) combined with atrial septal aneurysm (ASA) has a high incidence of cryptogenic stroke insufficiency, which is related to the size and activity range of atrial septal aneurysm^2^. ASA is a congenital atrial septal dysplasia disease. In recent years, some scholars believe that ASA is a potential source of cardiac thrombosis^3^–^4^. PFO refers to congenital heart disease, in which the oval foramen valve does not adhere to the secondary atrial septum after 3 years old, resulting in shunt at the atrial level^5^. Literature^6^ showed that the incidence of ischemic stroke in young and middle-aged PFO patients was significantly higher than that in patients without PFO, so PFO examination should be carried out as soon as possible. Correlation between cryptogenic ischemic stroke and atrial septal enlargement in China has been discussed. In order to explore the effects of ASA and PFO on the young and middle-aged patients with cryptogenic ischemic stroke, this study adopted the case-control study method and selected young and middle-aged patients with cryptogenic ischemic stroke in our hospital for study.

## Material and Methods

### General information

A case-control study was conducted to select 96 young and middle-aged patients with cryptogenic ischemic stroke (observation group) who were treated in the Department of Neurology of our hospital from January 2015 to January 2020, and 192 patients with non-ischemic stroke who were treated in the Department of Neurology of Tangshan Worker Hospital from January 2015 to January 2020 were selected as the control group. At the same time, 192 patients with non-ischemic stroke who were treated in the department of neurology were selected as the control group with gender and age matching of 1:2. There was no significant difference in gender and age between the two groups (P> 0.05) (Table 1).

**Table 1:** Comparison of gender and age between observation group and control group

Group	Case	male/female	Age (year)
Observation group	96	66/30	34.45±9.65
Control group	192	118/74	33.10±10.02
t/χ^2^		1.475	1.091
*P*		0.225	0.276

#### Inclusion criteria

(1) Age ≤ 55 years. (2) The diagnosis of cryptogenic ischemic stroke conforms to the definition of “Diagnostic guidelines for acute ischemic stroke in China” and Timsit^7^. (3) Patients in the observation group were treated within 48 h of onset and examined by transthoracic echocardiography (cTTE) in hospital. (4) The clinical image data were preserved completely.

#### Exclusion criteria

(1) A clear cause or suspicious explanation of the cause. (2) ther brain diseases such as cerebral hemorrhage, cerebral artery inflammation and hemorrhagic infarction.

### Imaging examination

Using the US-imported Philips IE33 ultrasonic system, each data is measured three times, and the average value is taken to ensure the minimum error. The probe frequency is 3.5–4.5 MHz. Esophageal ultrasound probe with 4.5 MHz color Doppler flow imaging have recorded. The frequency of the electronic phased array probe is set to 6.0 MHz, and the spectrum Doppler is set to 3.5 MHz. Esophageal echocardiography examination method is as follows : according to the conventional disinfection method of esophageal ultrasound probe disinfection, the probe front bend 140°∼60° forward, smear lubricant. The patient was asked to hold his breath on the left side. The esophageal ultrasound probe was inserted into the esophagus through the mouth and pharynx for scanning. The probe was scanned from 0° to 180° along the clockwise direction to observe whether the oval foramen flap on the double atrium was open, whether there was atrial septal aneurysm (ASA), and whether there was thrombosis in the atrial septal wall. In the resting state and Watt's action, whether there was a right-to-left shunt (RLS) in each patient was observed, and the average examination was 2 to 3 times.

### Diagnostic criteria

#### PFO diagnostic criteria^8^

There is a gap between the oval orifice valve and the second septum. In color Doppler flow imaging, there is a small atrial horizontal shunt between the secondary septum and the oval foramen valve, and the origin of the shunt is at the edge of the intersection between the secondary septum and the oval foramen valve. Two professional doctors judged the image. Two professional doctors judged the image. According to the diameter of the oval hole defect, PFO can be divided into large, medium and small, large ≥ 4.0 mm. Medium 2.0–3.9 mm. ≤ 1.99 mm is small. ASA diagnostic criteria^9^: Ultrasound showed local tumor-like processes ≥ 10 mm from the middle atrial septum to the atrial side.

#### Inspection indicators

Three milliliters fasting venous blood was collected, centrifuged at 3000 rpm/min for 5 min, and serum was separated. SF-8100 automatic coagulation analyzer was used to operate according to the instrument reagent and instructions. FIB, APTT, PT, Scr, TG, LDL-C and CRP levels were measured, and the detection should be completed within 2 h.

### Statistical analysis

SPSS22.0 software was used for data analysis. Normal distribution measurement data were expressed as (χ̅±s). T test was used for comparison between the two groups. Non-normal distribution measurement data were expressed as M (Q25, Q75). Rank sum test was used for comparison between the two groups. Count data were expressed as n (%), and the comparison between the two groups was performed using the χ_**2**_ test or Fisher exact test. Logistic regression analysis was used for multivariate analysis α=0.05 .

## Results

### Comparison of clinical general data between the observation group and the control group

FIB, APTT, PT, Scr, TG, LDL-C and CRP in the observation group were significantly higher than those in the control group (P<0.05), while AST was significantly lower than that in the control group (P<0.05) (Table 1).

### Comparison of cTTE results between observation group and control group

The PFO, PFO combined with ASA, and the right-to-left shunt II-III ratio and PFO diameter of cTTE in the observation group were significantly higher than those in the control group (P<0.05) (Table 2).

**Table 2:** Comparison of clinical general data between observation group and control group

Clinical data	Observation group (n=96)	Control group (n=192)	t/χ^2^	*P*
Body mass index(kg/m^2^)	24.10±2.06	23.89±2.15	0.792	0.429
Smoking(%)	68(70.83)	122(63.54)	1.516	0.218
Alcohol consumption (%)	54(56.25)	97(50.52)	0.842	0.359
Hypertension(%)	23(23.96)	44(22.92)	0.039	0.844
Diabetes(%)	12(12.50)	30(15.63)	0.502	0.479
Coronary heart disease(%)	8(8.33)	13(6.77)	0.231	0.631
Hyperlipidemia(%)	14(14.58)	20(10.42)	1.067	0.302
WBC(×10^9^/L)	6.60±1.06	6.84±0.95	-1.944	0.053
RBC(×10^12^/L)	4.56±0.82	4.60±0.90	-0.366	0.715
Hb(g/L)	140.41±15.54	139.89±12.26	0.310	0.757
FIB(g/L)	2.80±0.34	2.55±0.41	5.153	0.000
APTT(s)	34.41±5.31	32.20±4.89	3.513	0.001
PT(s)	16.60±1.65	14.84±1.80	8.038	0.000
ALT(U/L)	28.81±3.35	29.10±3.06	-0.734	0.463
AST(U/L)	24.46±2.03	25.50±1.95	-4.209	0.000
BUN(mmol/L)	4.52±0.64	4.32±0.71	2.327	0.021
Scr(µmol/L)	63.32±8.89	60.12±7.90	3.106	0.002
TC(mmol/L)	3.97±0.95	3.90±0.81	0.652	0.515
TG(mmol/L)	1.65±0.32	1.50±0.28	4.083	0.000
HDL-C(mmol/L)	1.10±0.27	1.14±0.30	-1.102	0.271
LDL-C(mmol/L)	2.40±0.65	2.21±0.71	2.201	0.029
CRP(mmol/L)	1.20±0.35	0.98±0.21	6.645	0.000

### Comparison of ASA between observation group and control group

There was no significant difference in ASA classification between the observation group and the control group (P>0.05). The ASA depth and tumor wall thickness in the observation group were significantly higher than those in the control group (P<0.05) (Table 3).

**Table 3:** Comparison of cTTE results between observation group and control group

Clinical data	Observation group (n=96)	Control group (n=192)	t/χ^2^	*P*
PFO	36 (37.50)	20 (10.42)	29.97	0.000
PFO combined with ASA	20 (20.83)	4 (2.08)	29.455	0.000
The ratio of right to left shunt II - III in Wagner maneuver cTTE	31 (32.29)	8 (4.17)	43.240	0.000
PFOdiameter (mm)	1.55±0.32	0.92±0.20	7.965	0.000

### Multi-factor analysis results

The above statistically significant indicators were used as independent variables and whether they were cryptogenic ischemic stroke as dependent variables for logistic regression analysis. The results showed that PFO, PFO combined with ASA were the influencing factors of cryptogenic ischemic stroke in middle-aged and young people (OR=1.923 and 1.384, P < 0.05) (Table 4).

**Table 4:** Comparison of ASA between observation group and control group

Group	cases	ASA		ASA depth (mm)	ASA Tumor wall thickness (mm)

		IB	IA		
observationgroup	20	16 (80.00)	4 (20.00)	15.50 (12.40,19.80)	3.90 (2.80,4.50)
controlgroup	4	4 (100.00)	0 (0.00)	12.10 (8.80,14.40)	2.80 (2.10,3.25)
Z		-		-5.654	-6.121
*P*		1.000		0.000	0.000

Note: Indicates the use Fisher accurate inspection.

## Discussion

Stroke in young and middle-aged adults usually refers to stroke in adults under 55 years of age. Compared with the elderly, the etiology and risk factors of ischemic stroke in young and middle-aged adults are more complex and diverse^10^. Affected by hemodynamics in circulation, the atrial septum will be slightly uplifted, but the depth of the uplift is small, and ASA diagnosis is difficult. At present, there is no gold standard for the diagnosis of ASA. It has been found that the local tumor-like projection towards a atrium ≥10 mm in the middle of the atrial septum is more suitable for the diagnosis of ASA^11^–^12^. The occurrence of ASA may be due to the pressure difference between the left and right atrium, or may be due to the weak development of fibrous connective tissue in the oval atrial septum, which leads to the lateral expansion of the atrial septum with low pressure^13^–^14^. In this study, there was no statistical significance between the two groups in the expansion of various types of ASA, indicating that the expansion direction and movement type of ASA had no significant effect on the occurrence of cryptogenic ischemic stroke. PFO is an abnormal channel in the atrial septum formed by the right atrial channel that cannot be normally closed after fetal childbirth, and the blood flows from right to left^15^. The mechanism of PFO-induced ischemic stroke is abnormal embolism: the pressure of the right heart is higher than that of the left heart. The embolus of the venous system returns to the right heart through the vena cava and does not pass through the pulmonary circulation and directly enters the left heart through the open oval foramen. Then it flows into the body circulation and blocks the intracranial artery^16^–^17^. In recent years, studies have found that PFO is closely related to neurological diseases, and its mechanism is considered to be related to abnormal embolism^18^–^19^.

The results showed that FIB, APTT, PT, Scr, TG, LDL-C and CRP in the observation group were significantly higher than those in the control group (P<0.05), while AST was significantly lower than that in the control group (P<0.05). It indicated that the above abnormal indicators could be used as a risk factor to participate in the occurrence of youth cryptogenic stroke. In the observation group, the proportion of PFO, PFO combined with ASA, and cTTE right-to-left shunt II – III and PFO diameter were significantly higher than those in the control group (P< 0.05). The results showed that PFO had a more important effect on the occurrence of ischemic stroke in young and middle-aged people. When the diameter of PFO was large or it was combined with ASA, it was more likely to cause ischemic stroke. When ASA combined with PFO, PFO shunts from right to left due to increased right heart pressure, and if peripheral venous thrombosis still exists, paradoxical embolism should be highly suspected in the event of cerebral ischemia^20^. In the present study, the proportion of patients with PFO in the observation group was also higher than that in the control group. The results suggest that effective Watt's maneuver is important in the diagnosis of PFO. It can be inferred that right-to-left shunt is related to stroke. The reason may be that the more shunts, the more likely the embolus enters the left heart circulation through the atrial septum, and the greater the risk of stroke. Identifying these auxiliary factors can predict high-risk populations and prevent stroke^21^.

The depth of ASA and the thickness of tumor wall in the observation group were significantly higher than those in the control group (P<0.05). This was due to the blood stasis in the ASA tumor, and the thickening, degeneration and damage of the intima of the ASA tumor wall, which was easy to platelet adhesion and thrombosis. In addition, ASA swing easily makes it fall off, causing embolism. In this study, the ASA tumor wall thickness of the observation group was significantly thicker than that of the control group, which did not exclude the possibility of microthrombosis. Logistic regression analysis was carried out with the above statistically significant indicators as independent variables and whether they were cryptogenic ischemic stroke as dependent variables. The results showed that PFO, PFO combined with ASA were the influencing factors of cryptogenic ischemic stroke in young and middle-aged people. Identifying these auxiliary factors can predict high-risk populations and prevent stroke. Since ASA plays an important role in cerebral ischemic lesions, attention should be paid to the existence and characteristics of ASA in patients, especially in patients with peripheral venous thrombosis. The shunt from right to left should also be found at the atrial level, whether PFO is combined, and if necessary, anticoagulant or surgical treatment should be sought. This study fills the gap that there is no research on the correlation between cryptogenic stroke and atrial septal aneurysm in China, and clarifies whether ASA combined with PFO is an independent risk factor for stroke, which helps clinicians to provide useful information and guidance in the diagnosis and treatment of cryptogenic stroke.

## Conclusion

ASA combined with PFO and PFO are independent influencing factors for the occurrence of cryptogenic ischemic stroke in young and middle-aged people, which is worthy of further study.

## Figures and Tables

**Fig. 1:**
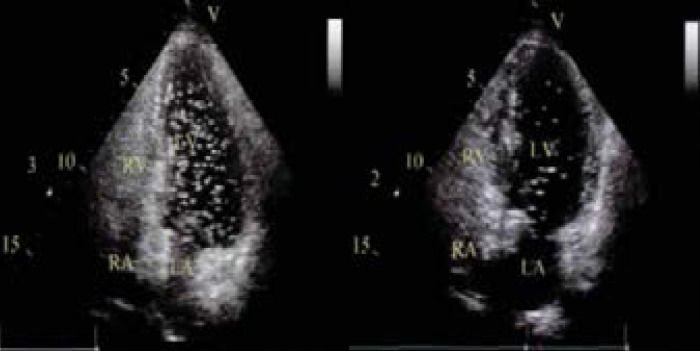
Unclosed cTTE Ovary Image A: Watts action cTTE right to left shunt class II; B: Proportion of right-to-left shunt stage III cTTE Watts movement

**Table 5:** Results of multivariate analysis

factor	β	SE	Walds	*P*	OR (95%CI)
PFO	0.654	0.168	15.154	0.000	1.923 (1.384∼2.673)
PFO combined with ASA	0.325	0.121	7.214	0.000	1.384 (1.092∼1.754)
Constant term	0.401	0.153	6.869	0.000	-
